# A quasi‐experimental study of the effect of teaching Orem's self‐care model on nursing students’ clinical performance and patient satisfaction

**DOI:** 10.1002/nop2.151

**Published:** 2018-04-23

**Authors:** Javad Malekzadeh, Zahra Amouzeshi, Seyed Reza Mazlom

**Affiliations:** ^1^ Department of Medical & Surgical Nursing Mashhad University of Medical Sciences Mashhad Iran; ^2^ Faculty of Nursing and Midwifery Birjand University of Medical Sciences Birjand Iran; ^3^ Isfahan University of Medical Isfahan Iran

**Keywords:** clinical training, nursing student, Orem's self‐care model, patient satisfaction, performance

## Abstract

**Aims:**

This study aims to determine the effect of teaching Orem's self‐care model on nursing students’ clinical performance and patient satisfaction.

**Design:**

The study was a quasi‐experimental, non‐randomized, two‐group design with posttest.

**Methods:**

In this quasi‐experimental study, 66 nursing students were selected via convenience sampling method. The intervention group was trained based on Orem's self‐care model and the control group based on the routine nursing process method. Both groups cared patients for a week. Students’ performance was evaluated during the clinical course by performance observation checklist and patient satisfaction was assessed at the end of clinical course using patient satisfaction form. Data were analysed in SPSS software using chi‐squared, Fisher Exact test, Mann–Whitney, *t* test and two‐way ANOVA.

**Results:**

Clinical performance evaluation mean score in the intervention group was significantly higher than that of the control group. However, patient satisfaction scores in both the control group and intervention group did not show statistically significant differences. Orem's self‐care model showed a 23% improvement in students’ performance. It is recommended to use Orem's self‐care model for undergraduate courses, especially in clinical training.

## 1 INTRODUCTION

Clinical training courses are considered as a central part of professional education. Based on some research findings, clinical nursing education faces problems such as decreasing efficacy, continuing traditional methods and resistance to innovation (Rahmani, Mohajjel Aghdam, Fathi Azar, & Roshangar, 2009; Sawatzky, Enns, Ashcroft, Davis, & Harder, 2009). One of the hindering underlying factors associated with quality and quantity care development lies with application of non‐pragmatic theoretical models. Orem's self‐care model leads to lower healthcare costs, quality care development and patient outcome improvement. Therefore, Orem's self‐care model of nursing can be used in the education of nursing students with the aim of bridging the gap between theory and practice (Timmins, 2002; Timmins & Horan, 2007; Wu, Chin, & Chen, 2009). The self‐care deficit nursing theory is comprised of three theories, that is, the theory of self‐care, the theory of self‐care deficit and the theory of nursing system. These three theories can be built on as the framework for the contents of a nursing education curriculum. Application of the theory of self‐care deficit as the conceptual framework for curriculum development is a more systematic approach to nursing education and can inspire a nursing‐based mode of thinking and communication (Hintze, 2011).

In Orem's self‐care model, nursing practices called “nursing systems theory” consist of wholly compensatory nursing system, partially compensatory nursing system and supportive‐educative nursing system. Therefore, the nurse employs one of the mentioned systems in the care plan according to the patient's abilities and limitations. In each of these systems, the agent can be acting/doing, assisting or guiding on the basis of the type of work she/he performs (Hashemi et al., 2014).

To prepare effective care and improve clinical education, it is important to evaluate the effect of teaching Orem's self‐care model both on the nursing students’ clinical performance and patients’ satisfaction with the care given by nursing students.

## 2 BACKGROUND

Caring, as the fundamental component of nursing, is widely accepted among nurses. Nurses’ caring aims to enhance patients’ well‐being and to facilitate health promotion (Khademian & Vizeshfar, 2008). To evaluate and improve the quality of care provided, it is of vital importance to investigate the quality of care in the healthcare services. In this line, patient satisfaction is a significant indicator or quality of care (Can, Akin, Aydiner, Ozdilli, & Durna, 2008; Johansson, Oleni, & Fridlund, 2002). It has been observed that evaluation of patients’ satisfaction with nursing care increases their adherence to medical regimens in patients and their regular visits for medical follow‐up after discharge (Can et al., 2008).

Nursing education and clinical care are closely connected (Yang et al., 2013). Nursing education plays an essential role in the ability to practice effectively. It follows that an optimally educated nursing workforce creates optimal patient care (Sawatzky et al., 2009).

In recent years, there has been a deep gap in the nursing education between theoretical and clinical education. Several studies indicate that nursing students, despite a good knowledge base, were not skilful in clinical settings. As a result, with entrance of these unskilled students to the clinical settings, the quality of these settings increasingly lowers down (Rahmani et al., 2009).

Nursing theories provide conceptual frameworks, offer instructions for nursing care and guide educators in the process of teaching nursing students (Wu et al., 2009). Orem's self‐care theory can work as a guide to nursing care in multiple settings and can involve patients throughout their lifespan in various stages of the health–illness continuum (Timmins, 2002). Orem's self‐care deficit nursing theory is widely accepted by nurses and used in clinical settings and is one of the most frequently used theories in general nursing practice (Timmins & Horan, 2007).

This theory can be related to the body of nursing knowledge in that it uses patients assessment skills, builds a nurse–patient relationship, plans how to meet the objectives of self‐care, implements interventions and evaluates how effective the interventions were to attaining health and self‐care along with changes as needed (Simmons, 2009). On the other hand, the self‐care deficit nursing theory is a curriculum conceptual framework in baccalaureate education as applied in Anderson University School of Nursing, Southern University Baton Rouge (SUBR) School of Nursing and University of Tennessee Chattanooga (UTC) School of Nursing (Berbiglia, 2011).

Silén and Johansson (2016) study in Sweden revealed that the theories/models more frequently used in nursing students’ bachelor theses comprise of Orem's self‐care theory, Travelbee's human‐to‐human relationship model and Eriksson's theory of caritative caring. Future research should cover the ways nursing and other nursing‐related theories are introduced during nursing programmes, the kinds of theories that are introduced and the kind of knowledge nursing students are provided with regarding how theories can be employed in independent projects (Silén & Johansson, 2016).

Research on the effects of this theory in nursing students’ clinical performance and patient satisfaction is scarce, but there are limited findings about the care provided by nursing students, especially about the satisfaction of patients with nursing students care. To prepare effective care and improve clinical education, it is important to evaluate the effect of teaching Orem's self‐care model both on the nursing students’ clinical performance and patients’ satisfaction with the care given by nursing students. This study had a single research question which is as follows:

What is the effect of teaching Orem's self‐care model on nursing students’ clinical performance and patient satisfaction?

## 3 THE STUDY

### 3.1 Design

The study was a quasi‐experimental, non‐randomized, two‐group design with posttest.

### 3.2 Method

The study population included all sixth semester, bachelor nursing students, who were in educational hospitals in Mashhad at the time of the research and who were willing to participate in the study. To determine the sample size, a pilot study was conducted with 20 students. The prediction of participant attritions in each group increased the sample size to 33 individuals. Hence, the study population included 66 nursing students who were selected using convenience sampling method and divided non‐randomly into two groups (intervention and control) after matching in terms of variables such as sex, trainer and training time (morning or afternoon). As concerns with other variables that could be confounding and were monitored, the results of statistical tests did not show a significant difference between the two groups in terms of the variables studied (age, sex, marital status of the student, clinical experience and its duration, mean scores of internship, GPA (Grade Point Average), interest in nursing, the entrance score in the national examination, the type of admission quota and the time lapse from obtaining diploma and entering the university); hence, they were homogeneous.

It should be mentioned that the students who were absent more than one day were excluded from the study and all participants were present throughout the research.

Data collection tools in this study included: (1) a researcher‐made questionnaire and (2) questionnaire of students’ awareness level of nursing process/Orem's self‐care model.

The researcher‐made questionnaire consisted of three parts including demographic characteristics form, checklist of students’ clinical performance [including 37 items regarding communication (10 items), caring (17 items) and educational (10 items) skills based on the scale of “Never,” “Rarely,” “Sometimes,” “Often” and “Always” scored from 0 to 4] and checklist of patient satisfaction [including 25 items regarding patients’ satisfaction with care given by nursing students in three domains of communication (4 items), caring (13 items) and educational (8 items) skills based on the scale of “Very low,” “low,” “medium,” “high” and “very high” scored from 1 to 5]. The total score of any student was obtained by the sum total score of the questionnaire presented from 0‐100 for purposes of easier expression and inference. Therefore, student performance was determined based on 0–100. In addition to the total score, the scores of each of the three performance areas were determined by the sum total scores of items related to that area, which were presented on a scale of 0–100. Patient satisfaction total score of student performance was obtained from the total sum of scores presented on a 1–100 scale for ease of expression and inference. In addition to the total score, the score of each of the three areas of satisfaction was obtained by the sum of items of that area presented out of 100. The questionnaire of student awareness of the nursing process/Orem's self‐care model includes two sections: 1) case introduction: demographic characteristics, patient medical history, signs and symptoms of the disease and physical examination; 2) 10 items: all of the items are multiple choice; only the first five items are related to case introduction. The true answer to each item received 1 score that is presented from 0 to 20 to become easier to explain. The validity of the researcher‐made questionnaire and the questionnaire of students’ awareness were confirmed by face validity using comments of 10 faculty members of School of Nursing and Midwifery of Mashhad. To assess the reliability of patient satisfaction checklist, Cronbach's alpha method was used, the obtained coefficients was 0.95. The reliability coefficient of the students’ clinical performance checklist was obtained at 0.80 using interrater method. The reliability of the questionnaire of students’ awareness of the nursing process and Orem's self‐care model were confirmed using Parallel‐Forms in a pilot study on 20 participants(*r* = .81 and *r* = .87, respectively .

The educational programme was presented by the researcher to the intervention group during a 3‐hr training session. In the control group, the nursing process was explained about 45 min. In educational session in both groups, lectures, questions and answers and clinical scenarios were used. At the end of the educational session in both groups, the awareness questionnaire was given to the students as posttest. Afterwards, the students had internship for a week and cared for patients. Students in both groups had the responsibility to care for a maximum of three patients during their clinical training under supervision of a supervisor. The method of care in the intervention group was based on Orem's self‐care model and in the control group according to the nursing process. Performance of students in both groups was assessed by the researcher based on performance observation checklist. The overall performance of each student was observed four times during care and at the end of the training. Also, the patients were interviewed for satisfaction of the care given by nursing students using Likert score.

### 3.3 Analysis

Data analysis was performed in SPSS 16 software (IBM Incorporation, Chicago, IL, USA). Quantitative variables normality was determined using the Kolmogorov–Smirnov test. Categorical variables were analysed using chi‐squared or Fisher exact test. Mann–Whitney, *t* test and two‐way ANOVA were used to compare continuous variables. The significance level was considered at *p* < .05.

### 3.4 Ethics

The protocol of the study was approved by the Research Department of Mashhad University of Medical Sciences under the identifier 88812.

## 4 RESULTS

The age range of students was between 20‐27 years. Most participants in the control and intervention groups were female with a ratio of 60.6% (female) and 57.6% (male) respectively. From among them, 33.3% in the two groups had clinical experience. Students’ interest in nursing was at a moderate level. According to their self‐report, 54.5% of the participants had never used the nursing process to care for their patients. Intervention and control groups were similar in terms of most variables. The differences found were related to the kind of training and gender of the patients. Most of the students in the control group (63.6%) and intervention group (45.5%) had passed their training in thoracic ward.

The mean score of awareness (out of 20) in the control and intervention groups was 13.3 (*SD* 3.0) and 12.2 (*SD* 3.0) respectively. The results of *t* test revealed that both groups were equal and that there was no significant difference in scores of the posttest. The mean performance score of students (out of 100) in three domains of communication, caring and educational skills in the intervention group was significantly higher than the control group (Table 1). The mean score of patient satisfaction (out of 100) in three domains of communication, caring and educational skills in both the control and intervention groups did not show statistically significant differences (Table 2).

**Table 1:** The comparison of mean scores of students in clinical performance in three domains in intervention and control groups

Group			
Domain	Intervention (*N* = 33)	Control (*N* = 33)	*P*
	Mean ± *SD*	Mean ± *SD*	
Communication skills	88.4 ± 10.6	79 ± 17.9	.014^a^
Caring skills	83.6 ± 10	69.2 ± 16.5	.001^b^
Educational skills	65.1 ± 15.8	42.5 ± 11.2	.001^b^
Total score	81.3 ± 9.82	66.1 ± 15.2	.001^b^

a Mann–Whitney.

b *t* test.

**Table 2:** The comparison of mean scores of patient satisfaction in three domains in intervention and control groups

Group			
Domain	Intervention (*N* = 30)	Control (*N* = 31)	*P*
	Mean ± *SD*	Mean ± *SD*	
Communication skills	87.0 ± 11.1	85.6 ± 13.9	.829^a^
Caring skills	79.8 ± 8.8	78.5 ± 12.5	.446^a^
Educational skills	47.0 ± 18.20	40.1 ± 15.4	.067^a^
Total score	70.5 ± 10.6	67.3 ± 10.3	.250^b^

a Mann–Whitney.

b *t* test.

Two‐way ANOVA test showed significant differences in clinical performance mean scores in terms of group and type of training (*p* < .001). However, group (*p* = .126) and type of training (*p* = .985) had no significant effect on clinical performance. There was not an interactional effect between these two variables on the clinical performance (*p* = .409). The two‐way ANOVA test results showed a significant difference in patient satisfaction according to group and gender (*p* < .001). While type of group had a significant impact on patient satisfaction (*p* = .020), patient gender had no significant effect on their satisfaction (*p* = .782). There was not an interactional effect between these two variables on patient satisfaction (*p* = .975).

## 5 DISCUSSION

The results of the present research showed that teaching Orem's self‐care model could improve students’ communication skills. Nurse–client interaction is the main area in collaborative nursing theories (Abdoli & Safavi, 2010). Orem's theory uses assessment skills, builds a nurse–patient relationship, plans to achieve the objective of self‐care, implements interventions and evaluates the extent to which the objectives of self‐care are achieved. Therefore, the first step is to establish a trustworthy nurse–patient relationship, interacting with the client to provide support and knowledge towards regaining self‐care (Simmons, 2009). In other words, communication skills if properly used can result in behaviour that can enhance awareness and contribute to attaining an acceptable level of nurse–patient relationship (Hazavehei, Moonaghi, Moeini, Moghimbeigi, & Emadzadeh, 2015). Patient assessments provide worthwhile feedback on the professional performance of students, aid students sense how they can influence patients’ situation and facilitate patients to get better outcomes (Salminen et al., 2010). Teaching Orem's self‐care model could improve students’ caring skills. Improvement of students’ communication skills can be one reason for improved care provided by them. It is because good communication with patients could help students to understand their problems more deeply and provide better care for them. Orem's self‐care model leads to lower healthcare costs, quality care development and patient outcome improvement (Richard & Shea, 2011). It can act as a guide to nursing practice in multiple settings and to improve the quality of nursing care. Also, self‐care is vital and profits both nurses and patients (Mills, Wand, & Fraser, 2015). In line with the goal of nursing practice, it assists patients to become adequately prepared to engage in their own care and thus, have improved patient outcomes and a better quality of life (Simmons, 2009). Actual relationships with patients are crucial in developing skills needed for the provision of proper nursing care (Salminen et al., 2010).

In the current study, teaching Orem's self‐care model could improve students’ educational skills. Given the emphasis in Orem's model on patient self‐care and independency, these two factors can contribute to the improvement of students’ educational performance. To have patient involvement in their own care, nurses must establish a trusting nurse–patient relationship, provide support and education system, allow patients some control of their situation by participating in decision‐making process and encourage patients to actively participate in the treatment (Simmons, 2009). Various studies indicate that good clinical communication has a positive influence on many outcomes ranging from patient satisfaction, consultation process and health behaviours, to human and economic costs of care (Perrona, Sommerc, Louis‐Simonetd, & Nendaz, 2015).

Teaching Orem's self‐care model on patient satisfaction in both the control and intervention groups did not show statistically significant differences. Intergroup comparison of the patients’ satisfaction scores showed that the intervention group satisfaction scores were higher in the three areas of communication, care and education, although this increase was not statistically significant. As this increase was related to all the three domains, it is not accidental. Therefore, it can be said that in case Orem's self‐care model was taught to students over a longer period, student performance and, consequently, patient satisfaction would enhance more considerably. By comparing the scores of the three domains, it can be said that from the perspective of patients, students’ educational skills are not in a good status as compared with other skills. The educational skills of students had a lower mean score from among the three domains of clinical performance. This is consistent with patient satisfaction score in terms of this domain. However, in Oskay, Güngör, and Basgöl (2015) and Can et al. (2008) studies, patients were highly satisfied with the care they received from the nursing students (Can et al., 2008; Oskay et al., 2015).

Satisfaction with the care provided by nursing students is important to ensure that nursing students, who will have primary responsibility for patient care in the future, receive good clinical and theoretical education and will enjoy improved clinical training (Can et al., 2008).

### 5.1 Limitations

However, an important point in clinical learning is practice. It seems that the current education has not been sufficient in this arena and it is essential that students have the opportunity to practice theories in clinical environment under the supervision of their educator. The facilities available in some wards were limited, inhibiting from holding training sessions. In addition, there are some factors that affect application of the new methods of teaching, including personal characteristics and differences in students in communicating with the clinical environment and their tendency to learn professional skills. Their personal characteristics and personality differences can affect patients in terms of satisfaction with the performance of students. Additionally, students may be affected by traditional methods for a long time. It should be mentioned that random selection of students was not possible since they were already allocated to apprenticeship groups by the faculty. Because of the non‐random sampling method in this study, findings may not be generalized to the general population. Therefore, it is recommended that a study with a random sample be conducted. If more time is spent for education according to Orem's self‐care model, greater impact would be exerted on students’ performance and hence greater satisfaction on the part of patients. Further research conducted with appropriate sampling and measurement of performance before and after the intervention is recommended. Also, it is suggested to have longer use of these models in clinical practice to assess long‐term impact of this model on student performance and patient self‐care. If the coaches assess students’ previous knowledge before the start of clinical work, more effective clinical education can happen given the fact that students’ weaknesses are known.

## 6 CONCLUSION

The results showed that each of the areas of clinical performance in the intervention group was significantly greater than that of the control group. Given the fact that Orem's self‐care model increased the performance of students, it can be stated that this model is more effective than the nursing process in improving the clinical performance of nursing students. Therefore, this model of nursing can be used in the education of nursing students with the aim of bridging the gap between theory and practice. By investigating patient satisfaction scores in the three areas of communication, caring and education in the two groups, it was determined that satisfaction scores in the intervention group overrode those of the control group although not statistically significant.

## CONFLICT OF INTEREST

The authors declare no conflict of interest in this study.

## AUTHOR CONTRIBUTIONS

JM substantially contributed to the conception and design; analysed and interpreted the data; drafted the article; approved the version to be published. ZA substantially contributed to the conception and design; involved in data acquisition, analysed and interpreted the data; drafted the article; approved the version to be published. SRM substantially contributed to the conception and design; analysed and interpreted the data; drafted the article; approved the version to be published.

## References

[nop2151-bib-0001] Abdoli, S. , & Safavi, S. S. (2010). Nursing students’ immediate responses to distressed clients based on Orlando's theory. Iranian Journal of Nursing and Midwifery Research, 15(4), 178. 21589792 PMC3093185

[nop2151-bib-0002] Berbiglia, V. A. (2011). The self‐care deficit nursing theory as a curriculum conceptual framework in baccalaureate education. Nursing Science Quarterly, 24(2), 137–145. 10.1177/0894318411399452 21471038

[nop2151-bib-0003] Can, G. , Akin, S. , Aydiner, A. , Ozdilli, K. , & Durna, Z. (2008). Evaluation of the effect of care given by nursing students on oncology patients’ satisfaction. European Journal of Oncology Nursing, 12(4), 387–392. 10.1016/j.ejon.2008.02.004 18653383

[nop2151-bib-0004] Hashemi, F. , Dolatabad, F. R. , Yektatalab, S. , Ayaz, M. , Zare, N. , & Mansouri, P. (2014). Effect of Orem Self‐Care program on the life quality of burn patients referred to Ghotb‐al‐Din‐e‐Shirazi burn center, Shiraz, Iran: A randomized controlled trial. International Journal of Community Based Nursing and Midwifery, 2(1), 40. PMCID: PMC4201185 25349844 PMC4201185

[nop2151-bib-0005] Hazavehei, S. M. M. , Moonaghi, H. K. , Moeini, B. , Moghimbeigi, A. , & Emadzadeh, A. (2015). Investigating the key factors in designing a communication skills program for medical students: A qualitative study. Electronic Physician, 7(7), 1441 10.19082/1441 26767096 PMC4700888

[nop2151-bib-0006] Hintze, A. (2011). Orem‐based nursing education in Germany. Nursing Science Quarterly, 24(1), 66–70. 10.1177/0894318410389057 21220579

[nop2151-bib-0007] Johansson, P. , Oleni, M. , & Fridlund, B. (2002). Patient satisfaction with nursing care in the context of health care: A literature study. Scandinavian Journal of Caring Sciences, 16(4), 337–344. 10.1046/j.1471-6712.2002.00094.x 12445102

[nop2151-bib-0008] Khademian, Z. , & Vizeshfar, F. (2008). Nursing students’ perceptions of the importance of caring behaviors. Journal of Advanced Nursing, 61(4), 456–462. 10.1111/j.1365-2648.2007.04509.x 18234042

[nop2151-bib-0009] Mills, J. , Wand, T. , & Fraser, J. A. (2015). On self‐compassion and self‐care in nursing: Selfish or essential for compassionate care? International Journal of Nursing Studies, 52(4), 791–793. 10.1016/j.ijnurstu.2014.10.009 25457876

[nop2151-bib-0010] Oskay, Ü. , Güngör, I. , & Basgöl, S. (2015). Evaluation of patients’ satisfaction with nursing students’ care on a perinatology ward. Journal of Nursing Education, 54(12), 696–703. 10.3928/01484834-20151110-06 26652805

[nop2151-bib-0011] Perrona, N. J. , Sommerc, J. , Louis‐Simonetd, M. , & Nendaz, M. (2015). Teaching communication skills: Beyond wishful thinking. Swiss Medical Weekly, 145, w14064 10.4414/smw.2015.14064 25664624

[nop2151-bib-0012] Rahmani, A. , Mohajjel Aghdam, A. , Fathi Azar, E. , & Roshangar, F. (2009). Comparison the effect of two clinical teaching models on performance of nursing students in intensive care unit. Iranian Journal of Nursing and Midwifery Research, 13(2).

[nop2151-bib-0013] Richard, A. A. , & Shea, K. (2011). Delineation of self‐care and associated concepts. Journal of Nursing Scholarship, 43(3), 255–264. 10.1111/J.1547-5069.2011.01404.x 21884371

[nop2151-bib-0014] Salminen, L. , Stolt, M. , Saarikoski, M. , Suikkala, A. , Vaartio, H. , & Leino‐Kilpi, H. (2010). Future challenges for nursing education–A European perspective. Nurse Education Today, 30(3), 233–238. 10.1016/j.nedt.2009.11.004 20005606

[nop2151-bib-0015] Sawatzky, J.‐A. V. , Enns, C. L. , Ashcroft, T. J. , Davis, P. L. , & Harder, B. N. (2009). Teaching excellence in nursing education: A caring framework. Journal of Professional Nursing, 25(5), 260–266. 10.1016/j.profnurs.2009.01.017 19751929

[nop2151-bib-0016] Silén, M. , & Johansson, L. (2016). Aims and theoretical frameworks in nursing students’ Bachelor's theses in Sweden: A descriptive study. Nurse Education Today, 37, 91–96. 10.1016/j.nedt.2015.11.020 26718541

[nop2151-bib-0017] Simmons, L. (2009). Dorthea Orem's self care theory as related to nursing practice in hemodialysis. Nephrology Nursing Journal, 36(4), 419. 19715109

[nop2151-bib-0018] Timmins, F. (2002). Critical care nursing in the 21st century. Intensive and Critical Care Nursing, 18(2), 118–127. 10.1016/S0964-3397(02)00006-X 12353650

[nop2151-bib-0019] Timmins, F. , & Horan, P. (2007). A critical analysis of the potential contribution of Orem's (2001) self‐care deficit nursing theory to contemporary coronary care nursing practice. European Journal of Cardiovascular Nursing, 6(1), 32–39. 10.1016/j.ejcnurse.2006.03.006 16713359

[nop2151-bib-0020] Wu, L.‐M. , Chin, C.‐C. , & Chen, C.‐H. (2009). Evaluation of a caring education program for Taiwanese nursing students: A quasi‐experiment with before and after comparison. Nurse Education Today, 29(8), 873–878. 10.1016/j.nedt.2009.05.006 19505747

[nop2151-bib-0021] Yang, W.‐P. , Chao, C.‐S. C. , Lai, W.‐S. , Chen, C.‐H. , Shih, Y. L. , & Chiu, G.‐L. (2013). Building a bridge for nursing education and clinical care in Taiwan—Using action research and Confucian tradition to close the gap. Nurse Education Today, 33(3), 199–204. 10.1016/j.nedt.2012.02.016 22480604

